# Postural Control Dysfunction and Balance Rehabilitation in Older Adults with Mild Cognitive Impairment

**DOI:** 10.3390/brainsci10110873

**Published:** 2020-11-19

**Authors:** Xuan Liu, Michelle H. Chen, Guang H. Yue

**Affiliations:** 1 Center for Mobility and Rehabilitation Engineering Research, Kessler Foundation, West Orange, NJ 07052, USA; XLiu@kesslerfoundation.org; 2 Department of Physical Medicine and Rehabilitation, New Jersey Medical School, Rutgers University, Newark, NJ 07101, USA; MChen@kesslerfoundation.org; 3 Center for Neuropsychology and Neuroscience Research, Kessler Foundation, East Hanover, NJ 07936, USA

**Keywords:** mild cognitive impairment, postural control, falls, balance rehabilitation

## Abstract

Older adults with mild cognitive impairment (MCI) are at an increased risk for falls and fall-related injuries. It is unclear whether current balance rehabilitation techniques largely developed in cognitively intact populations would be successful in older adults with MCI. This mapping review examined the available balance rehabilitation research conducted in older adults with MCI. Databases Medline, Cinahl, Cochrane, PubMed, Scopus, and PsycINFO were systematically searched from inception to August 2020. Twenty-one studies with 16 original randomized controlled trials (RCTs) involving 1201 older adults with MCI (>age 60) met the inclusion criteria, of which 17 studies showed significant treatment effects on balance functions. However, only six studies demonstrated adequate quality (at least single-blind, no significant dropouts, and intervention and control groups are equivalent at baseline) and evidence (medium or large effect size on at least one balance outcome) in improving balance in this population, and none of them are double- or triple-blind. Therefore, more high-quality RCTs are needed to inform future balance rehabilitation program development for older adults with MCI. Moreover, few studies examined the incidence of falls after the intervention, which limits clinical utility. Future RCTs should prospectively monitor falls or changes in risk of falls after the intervention.

## 1. Introduction

More than one in four adults aged 65 years and older fall each year [1,2]. Falls are the leading cause of fatal and non-fatal injuries among older adults [1,2]. In 2015, medical costs associated with older adult falls in the U.S. totaled USD 50 billion [3]. Cognitive impairment has been established as a significant risk factor for instability and falls among older adults [4]. Older adults with mild cognitive impairment (MCI) and dementia experience higher incidence of falls compared to cognitively intact older adults [5]. About 60% of older adults with MCI and dementia experience at least a fall every year, which is twice that of cognitively healthy older adults [6,7]. Cognitively impaired individuals are also more likely to experience injurious and non-accidental falls than their cognitively healthy peers [8,9].

MCI is a condition characterized by modest cognitive decline that does not yet significantly compromise everyday life independence. It is a reversible, transition stage between normal aging and dementia [10], with a 14.9% conversion rate to dementia among older adults with MCI monitored for two years [11]. In dementia, there is significant cognitive decline such that the individual is unable to complete daily activities without assistance. According to the most recent American Academy of Neurology (AAN) practice guidelines [11], rates of MCI steadily increase with age, with 8.4% in adults between the ages of 65 and 69 years to 25.2% of adults between the ages of 80 and 84 years. Although MCI was first conceptualized as a prodromal stage for Alzheimer’s disease, the current nomenclature refers to cognitive decline secondary to a variety of possible etiologies, including other dementing conditions (e.g., vascular dementia) as well as other neurologic and psychiatric disorders. MCI can be further categorized into amnestic (aMCI) and non-amnestic (nMCI) subtypes. aMCI is defined by predominantly memory dysfunction and more likely to represent a precursor to Alzheimer’s disease. nMCI refers to decline in primarily non-memory domains and can be the result of a myriad of conditions.

Increased fall risk among older adults with MCI and dementia is presumed to be the result of neurocognitive changes [12]. Specifically, decline in attention, mental processing speed, visuospatial abilities, and executive functions (i.e., higher-order cognitive abilities such as cognitive flexibility, planning, and behavioral inhibition) are associated with a higher risk of future falls, even after adjusting for other risk factors such as demographic variables and prior fall history [13,14]. Unsurprisingly, older adults with nMCI show a significantly higher fall risk compared to older adults with aMCI, which can be attributed to impaired executive functioning [8]. Declining cognition has been associated with more “risky” mobility activities (e.g., not holding onto a grab bar for support when stepping into the bathtub) and increased fall rate [15]. Thus, increased fall incidence in older adults with MCI may be partially attributed to judgment errors.

The interplay between cognition and mobility has been extensively studied using cognitive-motor dual-task paradigms (i.e., performing cognitive and motor tasks concurrently). Cognitive-motor dual-task paradigms have allowed researchers to investigate more subtle changes in gait and postural control related to cognitive decline among healthy older adults and older adults with MCI [16,17]. Patients with cognitive impairment often exhibit an increased “dual-task cost”, or a disproportionate decrement in performance under dual-task conditions compared to single-task conditions. Difficulties with dual-tasking are thought to reflect limited information processing capacity, and resource competition leads to performance decrement on one or both tasks [18]. The extent of dual-task difficulties depends on the severity of cognitive impairment, and older adults with MCI show a larger dual-task cost compared to healthy older adults [16,17]. Dual-tasking is an aspect of executive functions, which is often impaired in older adults with MCI. Importantly, poor dual-task performance, which is linked with conversion to dementia among older adults with MCI [19], is a significant predictor of future falls [5,13].

Postural instability is a major risk factor for falls [20,21]. Postural control worsens as a function of the degree of cognitive impairment, with older adults with MCI exhibiting an intermediate capability of postural control, between healthy older adults and individuals with dementia [20,22,23,24,25]. Larger postural sway while standing has been observed among older adults with MCI relative to healthy controls [22,23,26,27]. A recent meta-analysis revealed that anterior–posterior and mediolateral sway while standing during eyes open (but not eyes closed) best discriminates between older adults with MCI and healthy controls [28]. The authors hypothesized that impairment in visual processing among older adults with MCI may underlie this observed difference and recommended the use of visual feedback in balance rehabilitation.

The neural correlates of falls and postural control are not well understood. Extant literature indicates that a history of falls is associated with reduced gray and white matter volume, white matter lesions, and altered functional connectivity in both healthy older adults and older adults with MCI [29,30,31,32,33,34,35]. Brain regions involved in executive, visual, and motor functions (e.g., prefrontal cortex, pre- and post-central gyrus, occipital lobe) are particularly affected [29,32,34]. A decreased ability to deactivate brain connectivity during a task (i.e., increased functional connectivity in the default mode network, which is normally activated at rest but deactivated during a task) has also been observed in elderly fallers with and without MCI [31,33].

It is unclear whether balance rehabilitation techniques used in populations with predominantly motor difficulties would apply to individuals with cognitive impairment. Cognitive impairment may interfere with the ability to benefit from training or generalize training to activities of daily living. The MCI stage may be an ideal time to intervene, as it is a transition stage between normal aging and dementia. Once the older adult meets the criteria for dementia, there may be fewer options available for intervention given the extent of neurodegeneration and reduced ability to learn from training. Therefore, the current review will examine the available balance rehabilitation research conducted in older adults with MCI, which will inform future research and clinical care (e.g., incorporating balance training into standard of care for older adults with MCI).

## 2. Materials and Methods

A mapping review approach was selected to give an overview of the published randomized controlled trials (RCTs) which reported rehabilitation intervention effects on balance function in MCI. The purpose of this review was to identify gaps in the balance rehabilitation research for MCI and inform more specific future reviews and/or research studies on this particular area. The following databases were systematically searched from inception to August 2020: Medline (via EBSCOhost), Cinahl (via EBSCOhost), Cochrane, PubMed, Scopus, and PsycINFO (via Ovid). Searches used the following combination of keywords: (mild cognitive impairment) AND (exercise OR cognitive training) AND (balance OR postural OR fall).

Risk of bias was evaluated based on treatment allocation blinding (participants, assessors, and trainers), equivalency in baseline characteristics among treatment groups (or if differences were adequately accounted for in the analyses), and dropout rates [36]. Studies with significant dropout rates (>20%) [36] were noted in Table 1. Effect sizes (Hedges’ g) were calculated to determine the strength of the findings. For the effect sizes, pooled standard deviations from the experimental and control groups were weighted by sample sizes, and the baseline pooled standard deviations were used to calculate post-intervention effect size to minimize the influence of the interventions on the standard deviations [37]. Effect size was first calculated as the difference between the intervention and control groups at each time point (pre- and post-intervention). The difference between the two effect sizes were then derived as the final effect size for each comparison. Small sample sizes (total sample size [n] < 50) were corrected by multiplying the effect size with a bias correction factor ([n − 3]/[n − 2.25] × $\sqrt{\left[n-2\right]/n}$) [38]. Effect sizes were only calculated for studies purporting statistically significant group effects.

## 3. Results

The keyword search identified 655 articles, including 128 articles in EBSCOhost, 187 articles in Cochrane, 160 articles in PubMed, 98 articles in Scopus, and 82 articles in Ovid. After removing duplicates, 404 articles were checked for relevance based on the title and abstract using the following criteria: (1) the study sample included older adults with MCI; (2) a balance intervention was conducted; (3) the study design was RCT; (4) the outcomes included objective balance measures; (5) the article reported intervention results; (6) the article was written in English. The full texts of all articles that were deemed potentially relevant were then read by the authors using the same criteria. Additional articles were identified from references in relevant review articles that were found. The current review yielded a final sample of 21 RCTs.

### 3.1. Overview of Study Characteristics

#### 3.1.1. Year of Publication

The inception of balance rehabilitation studies in MCI was in the beginning of the last decade (Table 1). There has been an increase in published studies since 2011, with most studies published in 2016 (n = 5) [44,49,50,51,57] and 2019 (n = 4) [41,43,54,55].

#### 3.1.2. Sample Characteristics

There were a total of 16 original RCTs [39,40,41,42,43,44,46,47,49,50,53,54,56,57,58,59]; five studies [45,48,51,52,55] were secondary reports of the 16 RCTs (Table 1). Therefore, in the following sections, the study percentages demonstrating study outcomes and results (Section 3.1.7 and Section 3.2) were taken out of the 21 studies, while the study percentages describing other study characteristics (Section 3.1.3, Section 3.1.4, Section 3.1.5, Section 3.1.6 and Section 3.1.8) were taken out of the 16 original RCTs. A total of 987 older adults with MCI (age ≥ 60) were analyzed in those RCTs, with a mean sample size of 62 (range: 14 to 261) per trial. Nineteen percent (n = 3) [47,49,58] of the original RCTs recruited only older adults with aMCI, while the remaining 81% (n = 13) did not exclude older adults who had nMCI. In three original RCTs [39,43,44], cognitively intact older adults, individuals with Alzheimer’s disease, and individuals with Parkinson’s disease were additionally included as comparison groups.

#### 3.1.3. MCI Diagnosis

Forty-four percent (n = 7) [39,40,41,46,57,58,59] of the original RCTs used the Montreal Cognitive Assessment (MoCA) or a modified version of the MoCA as the primary criterion for MCI. Thirteen percent (n = 2) [44,53] used a score of 0.5 on the Clinical Dementia Rating (CDR) scale. Nineteen percent (n = 3) [42,49,56] established diagnosis through a clinical interview with a neuropsychologist or a neurologist-psychiatrist. Six percent (n = 1) [50] used criteria defined in the ICD-9-CM 331.83. Six percent (n = 1) [54] used the Addenbrooke’s Cognitive Examination (ACE) score. Six percent (n = 1) [47] used education-adjusted scores on the Wechsler Memory Scale-Revised (WMS-R) Logical Memory II. Six percent (n = 1) [43] used criteria defined in the Diagnostic and Statistical Manual of Mental Disorders (DSM-IV) (Table 1).

#### 3.1.4. Group Design

Most original RCTs (44%, n = 7) had a two-arm design comparing the physical training paradigm of interest with an alternative physical training paradigm (n = 2) [40,53] or a no-intervention control group (n = 5) [43,47,54,57,58] (Table 1). Thirty-one percent (n = 5) had a two-arm design comparing a combined physical and cognitive training group with a physical training group (n = 3) [41,44,50], a cognitive training group (n = 1) [49], or a no-intervention control group (n = 1) [46]. Thirteen percent (n = 2) [42,56] had a four-arm design, which included a combined physical and cognitive training group, a physical training group, a cognitive training group, and a no-intervention control or waitlist control group. Six percent (n = 1) [39] had a three-arm design, which included two combined physical and cognitive training groups with different levels of cognitive demand and a cognitive training only group. Six percent (n = 1) [59] had a three-arm design comparing the physical training paradigm of interest with two alternative physical training paradigms.

#### 3.1.5. Intervention Paradigms

A range of intervention paradigms and combinations of paradigms were used within the included RCTs (Table 1). The most common intervention paradigms used were combined physical and cognitive intervention (50%, n = 8) [39,41,42,44,46,49,50,56], followed by multimodal physical exercise (25%, n = 4) [43,47,54,56], Tai Chi (13%, n = 2) [53,58], ground kayak paddling exercise (6%, n = 1) [40], dumbbell exercise (6%, n = 1) [57], and elastic-band based exercise (6%, n = 1) [59].

#### 3.1.6. Intervention Dosage, Intensity, and Retention

Thirty-eight percent (n = 6) [40,41,44,46,49,50] of the original RCTs reported 6 to 10 weeks, 38% (n = 6) [42,43,56,57,58,59] reported 12 to 15 weeks, and the remaining 25% (n = 4) [39,47,53,54] reported 6 to 12 months of intervention (Table 2). All the RCTs included more than 12 sessions. Most RCTs included two training sessions per week (63%, n = 10) [40,41,42,43,46,47,49,50,54,59], 25% (n = 4) [44,53,57,58] included three sessions per week, 6% (n = 1) [56] included one to three sessions a week, and the remaining 6% (n = 1) [39] included two to five sessions a week (Table 2). Sixty-three percent (n = 10) [40,41,42,43,47,49,54,56,57,59] had training session durations from 60 to 90 min, and the remaining 38% (n = 6) [39,44,46,50,53,58] had session lengths fewer than 60 min (Table 2). Nineteen percent (n = 3) [44,55,59] designed a progressive increase in intensity during training (Table 2). Twenty-five percent (n = 4) included a follow-up session after the intervention program to assess for retention effects; among the four studies, three [42,44,56] designed a 6-month retest interval, while the remaining study [49] had a retest three months after the intervention (Table 2).

#### 3.1.7. Balance Outcomes

Fifty-two percent (n = 11) of the 21 studies (including both the original RCTs and the secondary reports) used Get-Up and Go (GUG, n = 1) [39] or a modified version Timed Up-and-Go (TUG, n = 9) [40,41,42,46,50,54,56,57,59] or 8-Foot Up and Go (8UG, n = 1) [43] test to measure dynamic balance and agility, which required subjects to stand up from a chair, walk a defined distance and back to the chair, and sit down again. Thirty-eight percent (n = 8) of the studies used functional reach test (FRT) (n = 4) [40,41,55,57], postural sway (n = 2) [41,49], one-leg stance (OLS, n = 2) [41,47], or functional stretching (n = 1) [50] to measure static or dynamic balance during standing under single task. Twenty-nine percent (n = 6) of the studies used the Berg Balance Scale (BBS, n = 5) [40,41,52,53,54] or the Balance Evaluation Systems Test (BESTest, n = 1) [51] to measure static and dynamic balance under multiple balance tasks. Nineteen percent (n = 4) used the Short Physical Performance Battery (SPPB, n = 2) [44,59] or the Tinetti Performance Oriented Mobility Assessment (Tinetti-POMA, n = 2) [46,50] to assess both mobility and balance under multiple physical tasks. Fourteen percent (n = 3) analyzed the number of falls (n = 3) [44,45,56] and/or calculated the Fall Rates to Activity (FRA) Index (n = 1) [45]; the FRA Index takes into account both changes in number of falls and daily walking activity. Ten percent (n = 2) [56,58] used the Physiological Profile Assessment to measure overall risk of falls through five sensorimotor subtests. Ten percent (n = 2) used the Fall Efficacy Scale-I (FES-I, n = 1) [50] or the Activities-Specific Balance Confidence (ABC) Scale (n = 1) [57] to measure the fear of falling. One study [41] used the Four Square Step Test (FSST) to assess the ability of subjects to step over low objects. One study [48] used harmonic ratio (HR) to measure the smoothness of trunk movement during walking.

#### 3.1.8. Risk of Bias

Six percent (n = 1) [53] of the original RCTs had a double-blind design, with the trainers and assessors blinded to the treatment allocation; participants were unblinded. Sixty-three percent (n = 10) [40,41,43,44,46,47,54,56,57,58] used a single-blind design, of which, all but one RCT blinded the outcome assessors from treatment allocation (the other RCT [54] blinded the statistician). Thirty-one percent (n = 5) [39,42,49,50,59] did not specify whether they masked the treatment allocation in intervention delivery or data analysis. All but one study reported no significant differences in baseline characteristics between the experimental and control groups, or accounted for baseline differences in their primary analyses. The remaining study [59] did not compare baseline characteristics in the manuscript (Table 1).

Twenty-five percent (n = 4) [39,43,53,59] of the original RCTs noted significant dropout rates (>20%) and differential dropout patterns among the treatment arms (one of the studies [39] changed their randomization scheme and had subjects self-select into the treatment arm with an especially high dropout rate). Although dropout rate was less than 20%, one study [49] reported that those who dropped out had significantly lower cognitive scores than those who stayed in the study (Table 1).

### 3.2. Study Results

Eighty-one percent (n = 17) [39,40,41,42,43,46,48,50,51,52,53,54,55,56,57,58,59] of the 21 studies reported beneficial effects of the targeted interventions on one or more balance outcomes, while 19% (n = 4) [44,45,47,49] reported no difference between the intervention and control groups in balance outcomes (Table 2). Negative effects of rehabilitation paradigms on balance function were not reported in any studies.

Of the 17 studies that reported significant intervention effects, four studies yielded small effect sizes (Hedge’s g ≈ 0.2) [39,52,53,57], three studies yielded medium effect sizes (Hedge’s g ≈ 0.5) [43,48,58], and four studies yielded large effect sizes (Hedge’s g ≈ 0.8 or larger) [40,50,54,55]. The remaining six studies [41,42,46,51,56,59] had variable effects sizes depending on the balance outcome and/or treatment group. Three studies [41,46,51] had a range of small to large effect sizes among their balance outcomes (in one study [51], the large treatment effects were primarily driven by pre-intervention differences rather than post-intervention differences between groups). One study found a medium effect in the physical training only group and a large effect in the combined physical and cognitive training group [42]. Another study found a small effect in the cognitive training only group and a medium effect in the combined physical and cognitive training group [56]. The last study yielded large effect sizes when the two intervention groups were compared against the control group, but the difference between the two intervention groups was small [59].

Only six studies [40,41,46,48,56,58] had at least the assessors blinded (single-blind), without significant dropout rates, and yielded a medium or large effect size on at least one balance measure. Choi and Lee (2018) [40] compared a ground kayak paddling exercise intervention (paddling while sitting on a chair following directions of trainers) with a home exercise control group. They found a large treatment effect in the ground kayaking group on the FRT relative to control. However, there were no significant group differences in other balance measures (TUG and BBS). The same research team followed up with a similar RCT, in which the intervention group performed kayak paddling exercises in a virtual environment (following movements of a pre-recorded kayak on a screen) [41]. In this study, statistically significant effects were found across all balance measures relative to control, and effect sizes ranged between small and large (with most in the medium range).

Delbroek et al. [46] reported that virtual reality physical and cognitive dual-task training on the BioRescue posturography platform significantly improved TUG performance, in comparison to a no-training control group. Three out of five subscores for TUG showed statistically significant effects (two with small or negligible effect sizes and one with a large effect size); five other reported measures were not statistically significant (Tinetti, four subscores for dual-task version of TUG). Another RCT compared a combined physical and cognitive training with physical training only, cognitive training only, and waitlist control [56]. No significant difference was found among groups in fall rate and PPA fall risk index post intervention, although TUG was improved in the combined training (medium effect compared to waitlist) and cognitive training only (small effect compared to waitlist) groups at follow-up. Although the study did not directly compare among the non-waitlist groups, our calculation of the effect sizes found small to medium effect (Hedges ≈ 0.4) sizes when comparing the combined training group to physical training only and cognitive training only groups. Of note, the effect seen in the combined versus cognitive groups was primarily driven by a pre-intervention difference between groups rather than post-intervention difference.

Doi et al. found a medium treatment effect on the smoothness of trunk movement using a multicomponent intervention program consisting of aerobic, strength, balance, and endurance exercises, compared to an educational control group among older adults with aMCI [48]. However, there were no statistically significant difference between the intervention and control groups on the OLS and a dual task with balance demand, which the authors attributed to the balance exercise being a small part of this multicomponent intervention program [47]. Finally, Sungkarat et al. found that Tai Chi significantly improved PPA fall risk index and postural sway relative to an educational control, with medium effect sizes, among older adults with aMCI [58].

## 4. Discussion

The aim of this mapping review was to evaluate the efficacy of balance rehabilitation research conducted in older adults with MCI on their balance function, in order to inform future research and clinical care. Twenty-one studies reporting on 16 original RCTs met the inclusion criteria and were analyzed. Four studies [47,48,49,58] included only older adults with aMCI, while the rest included mixed MCI subtypes. Studies varied in their determination of MCI diagnosis, but all used acceptable criteria, such as clinician diagnoses or validated cutoff scores on established neuropsychological measures. Although the majority of the studies reported beneficial effects on one or more balance measures, many studies suffered from a myriad of methodological limitations (e.g., inadequate masking of treatment allocation, significant dropout rates) that increased their risk of bias. Two-thirds of the RCTs had a single-blind design, with the outcome assessors blinded to treatment allocation. The only double-blind RCT blinded the trainers and assessors but not the participants; this trial also had significant dropouts which limited its validity [52,53]. The remaining RCTs did not specify any masking of treatment allocation, which made them highly biased. The gold standard for RCTs is to have a triple-blind design, where trainers, assessors, and participants are blinded to treatment allocation [36]. Understandably, this may be difficult to achieve in physical training interventions as opposed to a pharmaceutical trial. However, researchers should still use an active control group (instead of no treatment) and conceal study hypotheses from the participants and study personnel to minimize placebo effects and other sources of bias.

Moreover, many studies yielded small or variable effect sizes, potentially minimizing their clinical utility. Only six studies demonstrated adequate quality evidence, with at least a single (assessor)-blind design, no significant dropouts (>20%), and reported a medium or large effect size on at least one balance outcome measure [40,41,46,48,56,58]. Among these six studies, only two studies showed consistently high treatment effects above and beyond an active control group; one used a virtual kayaking intervention [41] and the other used Tai Chi [58]. That being said, neither of these studies masked the treatment allocation from the participants or the trainers, therefore increasing their degrees of bias.

Importantly, many studies did not report effect sizes, which limited the clinical interpretability of their results. In studies that did report effect sizes, most of them only calculated the post-treatment effect for each treatment arm separately, which did not elucidate whether the intervention of interest yielded a clinically relevant effect relative to control groups. The current review calculated effect sizes comparing the intervention and control groups, which often showed vastly different effect sizes from what the original trial papers reported. Taken together, more work is needed to establish an efficacious balance intervention for older adults with MCI. Future studies need to report the magnitude of treatment effects relative to control groups.

A major limitation of the literature base is the omission of falls in study outcomes. Only three studies (two original RCTs [44,56] and one secondary report [45]) measured the incidence of falls. None of these studies found a significant treatment effect in reducing falls relative to control in older adults with MCI. Thus, the currently limited literature has not demonstrated that balance interventions can reduce the fall incidence in older adults with MCI. Given the elevated risks of falls and fall-related injuries among older adults with MCI [5,6,8,9], more high-quality RCTs focusing on improving balance and reducing falls in this population is strongly needed. To accomplish this, prospective monitoring of falls after the intervention is recommended. However, because the number of falls need to be tracked prospectively and that may not always be easy to accomplish, examining the treatment effect on lowering the risk of falls in future studies may be a good alternative as long as the estimate of “risk of falls” is objective and accurate.

As mobility and cognition are inter-related based on common neural pathways [12], combining physical and cognitive training is a promising approach in balance rehabilitation for older adults with MCI. Only two studies utilizing a combined approach demonstrated adequate quality evidence [46,56]. Both studies showed highly variable effects on the balance outcomes included, each with multiple outcomes showing no statistically significant treatment effects. The probability for obtaining a type I error increases as the number of outcomes (thus number of tests) increases. In fact, in order to obtain the highest level of evidence (Class I) according to the AAN criteria for therapeutic trials, a RCT cannot have more than two primary outcome measures [36]. This prevents studies from examining a large number of outcome measures without a priori hypotheses on gold standard measures for the constructs of interest. Nevertheless, in the limited pool of adequate quality studies, it appears that combined physical and cognitive interventions may be more efficacious than physical or cognitive training alone. These will need to be confirmed with higher quality RCTs comparing a combined intervention with physical only and cognitive only control groups to elucidate the marginal efficacy of the combined intervention. Moreover, some of the studies we examined focused on cognition rather than balance as the primary construct of interest. Therefore, the researchers may not have designed the studies in order to maximize treatment effects on balance functions. Taken together, given the limited good quality RCTs in this area, future RCTs should target balance as the construct of interest with just one or two gold standard measures as primary outcomes, in order to detect true treatment effects.

In terms of training intensity, future studies may consider designing the interventions with increasing motor and cognitive demands, as older adults with MCI may not be able to tolerate high cognitive demands at the beginning of training. Retention effects should be examined after the interventions, as most studies did not conduct any long-term follow-ups. Possible mechanisms linking rehabilitation intervention to enhanced balance function were not demonstrated in the literature. Future studies may examine neuroimaging and/or electrophysiological outcomes before and after interventions to investigate possible neural mechanisms underlying the intervention-induced balance improvements.

## 5. Conclusions

The current mapping review identified 17 studies which showed significant treatment effects on improving balance function relative to control groups among older adults with MCI. However, only six studies demonstrated adequate quality (at least single-blind, no significant dropouts, and intervention and control groups are equivalent at baseline) and evidence (medium or large effect size on at least one balance outcome) in improving balance in this population, and none of them were double- or triple-blind. Therefore, more high-quality RCTs are needed to establish an efficacious balance intervention for older adults with MCI. Moreover, few studies examined training effects on the incidence of falls, which limits clinical utility. Future RCTs should prospectively monitor falls or changes in risk of falls after the intervention.

## Figures and Tables

**Table 1:** Sample characteristics, diagnosis, study design, and intervention paradigms in the RCTs included. Abbreviations: MCI mild cognitive impairment; MoCA Montreal cognitive assessment; DSM-IV diagnostic and statistical manual of mental disorders, fourth edition; CDR clinical dementia rating; MMSE mini-mental state examination; WMS-R Wechsler memory scale-revised; ICD-9-CM international classification of diseases, ninth revision, clinical modification; ACE Addenbrooke’s cognitive examination; ADL activities of daily living.

Author, Year	Sample Characteristics	Primary Diagnosis Criteria for MCI	Study Design	Training Intervention	Comparator Group (s)
Anderson-Hanley et al., 2018 [39]	14 MCI adherent at 6 month, enrollees (83 MCI and 28 cognitively intact), age > 65, predominantly female (66%)	MoCA < 26	3 groups: exer-tour (low cognitive demand), exer-score (high cognitive demand), and game-only (no physical exercise, not examined at 6 month); no blinding specified; 83% dropout rate; game-only group reported more sedentary lifestyle (due to high dropout rate for this non-exercising arm, researchers recruited specifically individuals who elected for this group)	exer-tour: virtual reality bike rides, exer-score: pedaling through a videogame	the same videogame operated by a joystick or keyboard
Choi and Lee, 2018 [40]	MCI (n = 60), age > 65, predominantly female (80%)	MoCA < 26	2 groups: ground kayak paddling and control; single (assessor)-blind	paddling exercise performed while sitting on chairs with and without a balance foam	a home exercise program
Choi and Lee, 2019 [41]	MCI (n = 60), age ≥ 65, predominantly female (85%)	MoCA < 26	2 groups: virtual kayak paddling and control; single (assessor)-blind	paddling exercise in a virtual environment	a home exercise program
Donnezan et al., 2018 [42]	MCI (n = 69), age > 65	diagnosed by a neuropsychologist with evidence of executive deficits	4 groups: simultaneous cognitive and physical training (PCT), physical training only (PT), cognitive training only (CT) or a no-intervention control group; no blinding specified	simultaneous cognitive and physical training: cognitive training and physical training delivered simultaneously within the same intervention	physical training: aerobic training on bikes, cognitive training: cognitive games, control: no intervention
de Oliveira Silva et al., 2019 [43]	19 MCI & 27 Alzheimer’s disease, age ≥ 65	structured clinical interview to assess mental disorders according to the DSM-IV	2 groups: exercise group, control group; single (assessor)-blind; 29% dropout rate in intervention group (7% in control group)	multimodal physical exercise (aerobic, strength, balance, and flexibility)	clinical follow-up without physical training
Mirelman et al., 2016 [44]	43 fallers with MCI, 109 older fallers, and 130 fallers with Parkinson’s disease, age 60–90, sex stratified	score of 0.5 on the CDR scale	2 groups: treadmill training plus virtual reality interventions, treadmill training only; single (assessor)-blind	subjects watched their feet projected on a screen via walking on a treadmill with real-life challenges (including obstacles, multiple pathways, and distractors) simulated	treadmill training alone
Del Din et al., 2020 [45]	38 fallers with MCI, 109 older fallers, and 128 fallers with Parkinson’s disease, age 60–90 (subset from Mirelman et al., 2016 [44]	score of 0.5 on the CDR scale	2 groups: treadmill training plus virtual reality interventions, treadmill training only; single (assessor)-blind	subjects watched their feet projected on a screen via walking on a treadmill with real-life challenges (including obstacles, multiple pathways, and distractors) simulated	treadmill training alone
Delbroek et al., 2017 [46]	17 institutionalized MCI, age ≥ 75	MoCA < 26	2 groups: intervention (i.e., virtual reality dual-task training using the BioRescue) or control group (no additional training); single (assessor)-blind	nine exercises which were used to train balance, weight bearing, memory, attention, and dual tasking	no intervention
Makizako et al., 2012 [47]	47 aMCI, age ≥ 65, female 46%	intact general cognitive function, MMSE 24–30, and having memory impairment (assessed via education-adjusted scores on the WMS-R Logical Memory II)	2 groups: multicomponent exercise or control group; single (assessor)-blind	combinations of aerobic exercise, endurance walking, muscle strength training, postural balance retraining, and gait training	attended two education classes about health promotion
Doi et al., 2013 [48]	47 aMCI, age ≥ 65, female 46% (same cohort as in Makizako et al., 2012 [47])	intact general cognitive function, MMSE 24–30, and having memory impairment (assessed via education-adjusted scores on the WMS-R Logical Memory II)	2 groups: multicomponent exercise or control group; single (assessor)-blind	combinations of aerobic exercise, endurance walking, muscle strength training, postural balance retraining, and gait training	attended two education classes about health promotion
Fogarty et al., 2016 [49]	41 aMCI, age > 60	based on an interview with the participant and an informant about history of cognitive concerns and functional decline, and a review of all available medical, neurological, psychiatric, and neuropsychological test data	2 groups: a combined Taoist Tai Chi and memory intervention group or memory intervention group; no blinding specified; dropout rates were <20% but those who dropped out had lower cognitive scores than those who completed the interventions	memory intervention plus a low-impact exercise program involving learning the practice of Taoist Tai Chi	a memory intervention program for MCI
Hagovska and Olekszyova, 2016 [50]	78 MCI, age 65–75	confirmed by their psychiatrist and psychologist and based on a standard clinical examination and neuropsychological testing, in line with the criteria defined in the ICD-9-CM 331.83	2 groups: CogniPlus or control group, no blinding specified	selected exercises from the CogniPlus program with balance training	daily balance training
Hagovska et al., 2016 [51]	78 MCI, age 65–75 (same cohort as in Hagovska and Olekszyova, 2016 [50])	confirmed by their psychiatrist and psychologist and based on a standard clinical examination and neuropsychological testing, in line with the criteria defined in the ICD-9-CM 331.83	2 groups: CogniPlus or control group, no blinding specified	selected exercises from the CogniPlus program with balance training	daily balance training
Lam et al., 2011 [52]	329 MCI, age > 65 (interim report for RCT described in Lam et al., 2012 [53])	CDR 0.5 or satisfying Mayo clinic criteria for aMCI	2 groups: mind body or control group; double (assessor and trainer)-blind	24-style Tai Chi	muscle stretching and toning exercises
Lam et al., 2012 [53]	261 MCI, age > 65	CDR 0.5 or satisfying Mayo clinic criteria for aMCI	2 groups: mind body or control group; double (assessor and trainer)-blind; significant dropout rates (46% in experimental and 22% in control)	24-style Tai Chi	muscle stretching and toning exercises
Langoni et al., 2019 [54]	52 sedentary MCI, age ≥ 60	medical records, home visits, and ACE score	2 groups: strength and aerobic exercises group or control group; single (statistician)-blind	strength: ankle weights, elastic bands, and dumbbells; aerobic: walking	no intervention
Langoni et al., 2019 [55]	52 sedentary MCI, age ≥ 60 (same cohort as in Langoni et al., 2019 [54])	medical records, home visits, and ACE score	2 groups: strength and aerobic exercises group or control group; single (statistician)-blind	strength: ankle weights, elastic bands, and dumbbells; aerobic: walking	no intervention
Lipardo and Tsang, 2020 [56]	92 MCI, age ≥ 60, female 79%	determined by a trained neurologist-psychiatrist based on 3 criteria: cognitive level is not normal nor demented, decrease in cognitive ability, and normal performance of basic ADL but with slight impairment in instrumental ADL	4 groups: combined physical and cognitive training, physical training, cognitive training, or waitlist control group; single (assessor)-blind	combined group: cognitive training elements incorporated in each type of exercise included in physical training group; physical training: multicomponent exercise program on balance, strength, endurance, and flexibility; cognitive training: paper-based cognitive exercises on executive function, memory, attention, and orientation training	no intervention
Lü et al., 2016 [57]	45 MCI, age ≥ 65	MoCA < 26, MMSE ≥ 24	2 groups: a dumbbell-training group (DTG) or a control group (CG); single (assessor)-blind	dumbbell-spinning exercises performed on the front part or lateral side of the body	no intervention
Sungkarat et al., 2017 [58]	59 aMCI, age ≥ 60, predominantly female (86%)	Petersen’s criteria, MoCA < 26, MMSE ≥ 24	2 groups: Tai Chi or control; single (accessor)-blind	3 weeks Tai Chi classes and 12 weeks practice at home	received educational material
Yoon et al., 2017 [59]	30 women with MCI, age > 65	Korean version of MoCA < 23	3 groups: an elastic band-base high-speed power training (HSPT), a low-speed strength training (LSST), or a control group; no blinding specified; significant dropout rates (30% in HSPT, 53% in LSST, and 63% in control); did not report whether baseline characteristics were equivalent among groups	both exercise regimens were based on the use of elastic exercise bands (the elastic band-base HSPT included a contraction phase instructed to be carried out as quickly as possible)	balance and tone exercises

**Table 2:** Training dosage, intensity, retention, balance outcomes, and balance related findings in the RCTs included. Abbreviations: MCI mild cognitive impairment; ES effect size (Hedge’s g); GUG get-up and go; TUG timed up and go; FRT functional reach test; BBS berg balance scale; OLS one-leg stance; FSST four square step test; 8UG 8-foot up and go test; ST_8UG single task in 8UG; CoV_8UG coefficient of variation of ST_8UG; DT_8UG dual task in 8UG; FRA falls rate to activity; SPPB short physical performance battery; HR harmonic ratio; FES-I fall efficacy scale-international; BESTest balance evaluation systems test; PPA physiological profile assessment; ABC activities-specific balance confidence.

Author, Year	Training Duration	Training Intensity Progression	Training Frequency	No. of Sessions	Length of Intervention Program	Retention Interval	Balance Outcomes	Balance Related Findings
Anderson-Hanley et al., 2018 [39]	20–45 min	n/a	2–5×/week	n/a	6 months	n/a	GUG	Performance in GUG increased significantly more in exer-tour compared with exer-score group (ES = 0.31).
Choi and Lee, 2018 [40]	60 min	n/a	2×/week	12	6 weeks	n/a	TUG test, FRT, and BBS	All balance outcomes were improved in both groups. The ground kayak paddling exercise was more effective for improving FRT than the control (ES = 1.08).
Choi and Lee, 2019 [41]	60 min	n/a	2×/week	12	6 weeks	n/a	ML/AP postural sway and velocity moment with eyes open/closed (EO/EC), OLS, TUG, FRT, BBS, and FSST	All balance outcomes were significantly improved in the virtual kayak paddling group and were superior to those in the control (EO-MLS ES = 0.63; EO-APS ES = 0.6; EO-VM ES = 0.39; EC-MLS ES = 0.51; EC-APS ES = 0.52; EC-VM ES = 0.38; right OLS ES = 1.03; left OLS ES = 0.77; TUG ES = 0.57; FRT ES = 0.44; BBS ES = 1.00; FSST ES = 0.63).
Donnezan et al., 2018 [42]	60 min	n/a	2×/week	24	12 weeks	only training groups evaluated at 6-month retest	TUG	TUG improved after physical training (ES = 0.50 vs. control) and simultaneous cognitive and physical training (ES = 1.29 vs. control). Retention observed at 6 months in the combined training group.
de Oliveira Silva et al., 2019 [43]	60 min	n/a	2×/week	24	12 weeks	n/a	ST_8UG, CoV_8UG, DT_8UG, DTC_8UG	The exercise program improved ST_8UG more than the control in MCI (ES = 0.61). No differences between training groups in individuals with Alzheimer’s disease.
Mirelman et al., 2016 [44]	45 min	motor and cognitive challenges increased based on subjects’ performance	3×/week	18	6 weeks	6 months	fall rates and fall status (whether a subject had ≥ 2 falls), SPPB	Fall rates and fall status improved similarly in treadmill training and treadmill training plus virtual reality groups in MCI.
Del Din et al., 2020 [45]	40 min	n/a	3×/week	18	6 weeks	6 months	number of falls, FRA	Both treadmill training and treadmill training plus virtual reality groups reduced FRA in MCI at 6 months post-intervention without between-group interaction.
Delbroek et al., 2017 [46]	gradually increased from 18 min in week 1 to 30 min in week 5	n/a	2×/week	12	6 weeks	n/a	iTUG, iTUG+DT, Tinetti-POMA	The intervention group improved significantly on iTUG total duration (ES = 0.05), turn-to-sit duration (ES = 0.19), and step-time before turn (ES = 1.15). No changes over time for either group in iTUG+DT or Tinetti-POMA.
Makizako et al., 2012 [47]	90 min	n/a	2×/week	n/a	6 months	n/a	OLS, dual task performance with balance demand	There were no significant improvement effects on balance-related outcomes.
Doi et al., 2013 [48]	90 min	n/a	2×/week	n/a	6 months	n/a	HR	The intervention group had a significant effect on the vertical HR (ES = 0.64).
Fogarty et al., 2016 [49]	90 min	n/a	2×/week	20	10 weeks	3 months	postural sway on regular/disturbed surfaces with eyes open/closed	No significant change in postural sway for combined Taoist Tai Chi and memory intervention group compared with memory intervention only group.
Hagovska and Olekszyova, 2016 [50]	30 min	n/a	2×/week	20	10 weeks	n/a	FES-I, Tinetti-POMA, and functional stretching, TUG	The experimental group showed better performance after training in Tinetti-POMA (ES = 0.89) and TUG (ES = 0.87) with dual tasking.
Hagovska et al., 2016 [51]	30 min	n/a	2×/week	20	10 weeks	n/a	BESTest	The experimental group showed better performance after training in BESTest postural reaction (ES = 0.25) and total score (ES = 0.88; largely accounted for by pre-intervention rather than post-intervention differences between groups). 5 other statistically insignificant subscores.
Lam et al., 2011 [52]	30 min	n/a	3×/week	n/a	1 year	n/a	BBS	The intervention group showed improved BBS score 2 months after completing induction (the induction phase lasted 8-12 weeks; ES = 0.13).
Lam et al., 2012 [53]	30 min	n/a	3×/week	n/a	1 year	n/a	BBS	The intervention group had better performance over the control group at 1 year (ES = 0.28).
Langoni et al., 2019 [54]	60 min	n/a	2×/week	48	6 months	n/a	BBS, TUG	The training group showed improvement in BBS (ES = 1.08) and TUG (ES = 0.91) after intervention.
Langoni et al., 2019 [55]	60 min	increased in strength exercise on incremental number of sets and repetitions and subjects’ performance	2×/week	48	6 months	n/a	FRT	The training group showed improvement in FRT after intervention (ES = 0.74; control group declined while training group improved).
Lipardo and Tsang, 2020 [56]	60–90 min	n/a	1–3×/week	n/a	12 weeks	6 months	fall rate, PPA, TUG	No significant difference among groups on fall rate and PPA score post intervention. TUG improved in cognitive training group (ES = 0.21 vs. waitlist) and combined physical and cognitive training group (ES = 0.55 vs. waitlist; ES = 0.4 vs. physical only; ES = 0.38 vs. cognitive only (primarily accounted for by pre-intervention difference between groups)).
Lü et al., 2016 [57]	60 min	n/a	3×/week	36	12 weeks	n/a	TUG, FRT, ABC	The intervention improved TUG performance compared with control (ES = 0.25).
Sungkarat et al., 2017 [58]	50 min	n/a	3×/week	n/a	15 weeks	n/a	PPA, PPA parameter scores (e.g., postural away)	Tai Chi group significantly improved PPA score (ES = 0.56) and PPA parameter scores (e.g., postural sway, ES = 0.59) post-intervention than control.
Yoon et al., 2017 [59]	60 min	progressively increased with time	2×/week	24	12 weeks	n/a	SPPB, TUG	SPPB increased significantly in high-speed power training (HSPT) and low-speed strength training (LSST) groups compared with control (ES = 0.89 for HSPT, ES = 0.70 for LSST). HSPT resulted in higher changes in SPPB and TUG versus LSST (ES = 0.36 for SPPB, ES = 0.35 for TUG).
